# Monozygotic twins with early-onset schizophrenia and late-onset bipolar disorder: a case report

**DOI:** 10.1186/1752-1947-7-134

**Published:** 2013-05-28

**Authors:** Richard O’Reilly, E Fuller Torrey, Jay Rao, Shiva Singh

**Affiliations:** 1 Department of Psychiatry, University of Western Ontario, St. Joseph’s Regional Mental Health Care, 850 Highbury Avenue North, London, ON, N6A 4H1, Canada; 2 The Stanley Medical Research Institute, 8401 Connecticut Avenue, Suite 2000, Chevy Chase, MD, 20815, USA; 3 Department of Biology, Biological and Geological Sciences Building, University of Western Ontario, 1151 Richmond Street, London, ON, N6A 5B7, Canada

**Keywords:** Schizophrenia, Bipolar disorder, Identical twins, Genetics

## Abstract

**Introduction:**

Schizophrenia and bipolar disorder are generally considered to be distinct illnesses. One piece of evidence supporting their distinctness is the rarity of schizophrenia and bipolar disorder occurring in monozygotic co‒twins.

**Case presentation:**

We describe a well-characterized pair of African American, female, monozygotic twins assessed at 53 years of age.

Case 1: Twin A developed psychotic symptoms at age 23. She was hospitalized and diagnosed with schizophrenia. Twin A was subsequently hospitalized several more times and was consistently diagnosed as suffering from schizophrenia. At the time of assessment, Twin A was single, lived with her parents and attended a day program. Case 2: In contrast, Twin B worked in a professional career, married and raised a family. She remained well until age 48 when she developed a depressive disorder requiring medication treatment. Four years later, Twin B abruptly developed grandiose delusions and mood-congruent auditory hallucinations. She was hospitalized and diagnosed with a manic episode. Since then Twin B has remained symptom-free on the mood stabilizer sodium valproate.

**Conclusion:**

Schizophrenia and bipolar disorder can occur in identical co-twins. We speculate on what it tells us about the meaning of discordance and the putative role of *de novo* mutations.

## Introduction

Emil Kraepelin is credited with differentiating dementia praecox from manic depressive illness. Kraepelin used the term dementia praecox to describe an illness characterized by hallucinations and delusions that began in adolescence or early adult life and was accompanied by a decline in function over time. In contrast, Kraepelin saw manic depressive illness as characterized by periodic episodes of depression and manic excitement with a return to normal functioning between episodes. Subsequent authors redefined the concept of dementia praecox, notably Eugene Bleuler who coined the term schizophrenia. Bleuler viewed schizophrenia as a group of illnesses rather than a single entity and expanded the Kraepelinian concept by including patients with a later onset and patients with less severe functional decline.

Formal diagnostic systems developed during the 20th century, such as the International Classification of Diseases and the Diagnostic and Statistical Manual of Mental Disorders (DSM), tinkered with the diagnostic criteria for both schizophrenia and manic depressive illness, but maintained the basic division. These diagnostic classification systems now refer to manic depressive illness as bipolar disorder or bipolar affective disorder. Despite the distinctive diagnostic criteria for the two disorders, clinicians regularly encounter patients who have admixtures of schizophrenic and bipolar symptoms. Consequently, diagnostic systems were forced to add the hybrid category: schizoaffective disorder. Some clinicians have argued that the inability to completely separate schizophrenia and bipolar disorder indicates that these illnesses are not discrete entities, but lie on a continuum [1].

In favor of the continuum hypothesis are the observations that unipolar depression occurs more frequently in the offspring of probands with schizophrenia [2], and that schizophrenia is overrepresented in the relatives of probands with bipolar disorder [3]. Also, a recent meta‒analysis of family studies found evidence of family coaggregation of schizophrenia and bipolar disorder [4]. However, other studies suggest that schizophrenia and bipolar disorder tend to breed true [5].

Monozygotic twin pairs in which one twin has schizophrenia and the other bipolar disorder would also be more consistent with the continuum hypothesis. However, reports of such twin pairs in the literature are rare [6], suggesting that an identical set of genes does not confer liability for both illnesses. In this article, we briefly review the few cases in the literature that report schizophrenia and bipolar disorder occurring in twins and triplets and describe a new case.

Our literature review identified one set of identical triplets and two pairs of monozygotic twins that were discordant for type of psychosis. Zygosity in the triplets was confirmed by multiple genetic markers [7]. Clinical diagnosis in the triplets was made on the basis of standardized interviews supplemented by a review of case notes. Written case summaries of the three individuals and additional decoy subjects were also rated blindly by assessors. On the basis of these assessments, two individuals were diagnosed with schizophrenia and one with manic depressive illness although the authors noted that there was some inconsistency and overlap in diagnosis depending on the type of testing.

Subsequently Dalby *et al*. [8] reported a monozygotic twin pair in which the determination of zygosity was made on the basis of physical similarity. The diagnosis of schizophrenia and bipolar disorder in the respective twins was made independently by a psychiatrist and a psychologist.

In the second reported pair of monozygotic twins discordant for psychosis [9], zygosity was determined by blood group analysis and diagnosis was determined on the basis of the Schedule for Affective Disorders and Schizophrenia (SADS) and the DSM‒III.

One twin was diagnosed as suffering from either chronic paranoid or chronic undifferentiated schizophrenia during several hospitalizations and on the basis of the SADS interview. The diagnosis of the second twin was less clear. The clinical diagnosis was schizoaffective disorder - whereas a diagnosis of manic depression was made on the basis of the SADS interview.

## Case presentation

Here we describe an additional pair of monozygotic twins, in which each co‒twin had a rigorous independent psychiatric assessment for two separate research studies conducted 22 years apart and were found to be discordant for both type of psychosis and age of onset.

The female twins were of African American ethnicity and were the first-born children to a 21-year‒old woman and were delivered vaginally after an uneventful pregnancy. Twin A was a breech delivery and weighed 4lb 11oz, while twin B was a vertex delivery and weighed 4lb 13oz. Developmental milestones were reached within the normal age range. Twin B had a tonsillectomy at age 18, otherwise, neither twin had significant childhood medical illness. Both twins completed high school following which they obtained degrees at separate colleges. There was no family history of affective or psychotic illness in first-degree relatives of the twins.

### Case 1

Following her graduation from college, Twin A went to work in the service field. Her family reported that for six to nine months before the onset of psychotic symptoms Twin A displayed prodromal symptoms such as irrational fears and ‘premonitions’. Twin A had an abrupt onset of inappropriate giggling and bizarre behavior at age 22. She was hospitalized and noted to have awkward posturing and mutism. She was withdrawn and suspicious and expressed suicidal ideation. The initial diagnosis was catatonic schizophrenia with paranoid features. She was treated with antipsychotic medications for three days and discharged to her parents’ home. Two weeks later, she left her parents’ home and shortly after was admitted to a psychiatric unit in a nearby town. She was noted to be confused and to have loose associations. She was diagnosed as having an acute schizophrenic reaction and treated with antipsychotic medication. During the next seven years, she had approximately 10 additional admissions. Her symptoms included auditory, visual, olfactory and somatic hallucinations. She also experienced incoherent speech, flight of ideas, echolalia, thought blocking, flat affect, bizarre behavior and both grandiose and paranoid delusions. She was consistently diagnosed as having schizophrenia, except on one occasion when she was diagnosed as having manic depression and given a trial of lithium. Twin A relapsed several times on this regime and it was discontinued in favor of restarting antipsychotics. She was treated with long‒acting injections of fluphenazine decanoate for six years before being placed on clozapine. A year later, sodium valproate was added to the treatment when she developed episodes of mildly euphoric and depressed mood. At the time of the second research assessment, Twin A was 53, living with her elderly parents and attending a day program.

### Case 2

Following graduation from college, Twin B got a job in a professional field. She married at age 31 and continued to work for 15 years until she emigrated to another country. Twin B had no symptoms of mental illness until she reached the age of 48 when she experienced a depressive episode characterized by low mood, decreased interest, insomnia, poor concentration and suicidal ideation. Twin B was treated with the antidepressant fluoxetine. Twin B took fluoxetine for more than four years. During that time Twin B’s symptoms fluctuated and she concluded that the antidepressant medication was not helping and stopped the medication herself. Two weeks after stopping fluoxetine, Twin B became euphoric. She started to get up earlier each morning. Although she had never written before, Twin B suddenly started to write a novel. She developed flight of ideas and displayed rapid speech. She jumped from topic to topic when speaking and made associations based on word similarities. Twin B was admitted to a psychiatric unit when she developed grandiose delusions and heard voices telling her the plot of the book she should write. Twin B was assessed by a psychiatrist who diagnosed a bipolar disorder, with a manic episode. She was treated with a combination of sodium valproate and olanzapine. Twin B responded well to this combination and was discharged from hospital after four weeks. The olanzapine was later stopped and when last assessed Twin B continued to take sodium valproate and remained well.

This twin pair was exceptional in that they had two comprehensive diagnostic assessments for research studies. The twins were first assessed by one of us (EFT) when they were 31 years old and participated in the National Institute of Mental Health (NIMH) schizophrenia and bipolar disorder twin study [10]. The twins were reassessed 22 years later (by ROR) for a second research study. The NIMH study used a consensus diagnosis by two senior psychiatrists (including EFT) who conducted a videotaped Structured Clinical Interview for DSM‒III‒R. Past psychiatric records, an interview with the twin’s mother, the results of a Minnesota Multiphasic Personality Inventory (MMPI) and informal observations made during testing were also considered in determining the diagnosis for this study. Twin A was diagnosed as having undifferentiated schizophrenia and Twin B did not have evidence of an Axis I disorder. During the second study 22 years later, the twins were assessed by the first author using the Structured Clinical Interview for DSM‒IV. The interview was videotaped and independently reviewed by a second senior psychiatrist (JR). An updated clinical record was considered in coming to a final diagnosis. Both psychiatrists in the second study concluded that Twin A was suffering from paranoid schizophrenia and Twin B had a bipolar I disorder in remission. Monozygosity of this twin pair was confirmed during the NIMH study by testing 19 red blood cell antigens. It was reconfirmed for the second study using 72 highly polymorphic single nucleotide polymorphism (SNP) probes on the Affymetrix® Human SNP Array 6.0 (Affymetrix Inc., Santa Clara, CA, USA).

## Discussion

The clinical features associated with this twin pair tested during their early 30s and again in their early 50s appear to provide the most reliable diagnoses of schizophrenia and bipolar disorder occurring in monozygotic co‒twins. However, even in this pair, there is symptomatic overlap similar to that described in previously reported cases. While Twin A was diagnosed with schizophrenia, she had exhibited occasional euphoria and sodium valproate was eventually added to her treatment regime. Conversely, when Twin B developed a manic episode, she exhibited mood-congruent hallucinations and delusions. This raises two questions about the meaning of disconcordance: what type and degree of psychopathology is permissible in a ‘well’ twin, and how much time must pass after the first twin becomes ill before we can confidently declare a twin pair to be discordant?

The twins described here are concordant for psychotic illness but discordant for schizophrenia and bipolar disorder. This twin pair highlights the difficulty inherent in classifying twins as concordant or discordant when the well twin has significant psychopathology. A diagnosis of schizophrenia in one twin and schizotypal personality disorder in the other would lead most researchers to classify a twin pair as concordant because of evidence of a genetic link between schizophrenia and schizotypal personality disorder. But how should researchers classify a pair in which the index twin has schizophrenia and the co‒twin a severe obsessive compulsive disorder?

The time interval between when Twin A became ill and Twin B displayed significant psychopathology indicates discordance for illness onset. In the cases previously reported in the literature, all siblings became ill within five years of each other. This is consistent with the observation that there is a significant correlation for the age of onset of twins who are concordant for schizophrenia [10,11]. In the pair reported here, Twin A developed psychotic symptoms at 22 and it was an additional 26 years before her sister exhibited significant psychopathology and 30 years before she exhibited psychotic symptoms. An interval of 31 years between the onset of schizophrenia in monozygotic twins [12] has been previously reported. While it is never possible to be sure that any twin pair will remain discordant, follow‒up studies of identical twins discordant for schizophrenia report that the majority of twins who become concordant do so within five years following the onset of schizophrenia in the first twin [11].

The cause of discordance for the symptoms and age of onset of psychosis in monozygotic twins is unknown. The heritability of schizophrenia has been calculated as approximately 80% [13] indicating that most of the variance is caused by genetic factors. Neither of the two twins described here experienced any of the putative environmental causes of schizophrenia such as perinatal trauma, intrauterine infection or head injury. Nor had they displayed a significantly different pattern of street drug use.

Researchers have identified a large number of putative causative genes for both schizophrenia and bipolar disorder. Some of these genes appear to predispose to both illnesses [14], but no gene appears to be necessary or sufficient to cause either illness. The current thinking is that many genes are likely required to reach a threshold where the full phenotype of either disorder is expressed.

Recent reports suggest that *de novo* mutations play a role in sporadic cases of schizophrenia [15]. We have proposed that *de novo* mutations also explain disease discordance observed in monozygotic twins [16] and note that *de novo* mutations can provide an explanation for the discordance for both the type and timing of onset of psychosis seen in this pair. Thus, both Twin A and Twin B may have inherited a mix of genes predisposing both of them to develop a mild bipolar disorder in later life. Then a *de novo* mutation occurring in early development in Twin A could have diverted her clinical trajectory so that she developed schizophrenia. Dramatic advances in total genome sequencing may now allow us to detect *de novo* mutations and to determine if they occur in pathways that could cause the psychopathology seen in schizophrenia and bipolar disorder.

Epigenetic mechanisms may also contribute to the threshold to develop schizophrenia and to the timing of onset. A possible explanation for discordance for both disease and age of onset is that two individuals develop epigenetic differences throughout their lives after they separate from a single zygote [17]. In support of this possibility we note that Fraga reported that methylation patterns are similar in monozygotic twins at three years of age but very different at age 50 [18].

## Conclusions

Schizophrenia and bipolar disorder do occur in well-characterized monozygotic co‒twins. Analysis of the genomes of monozygotic twins discordant for symptoms has the potential to identify genes contributing to the development of schizophrenia and related disorders.

## Consent

Written informed consent was obtained from each of the twins for publication of this manuscript. A copy of the written consent is available for review by the Editor‒in‒Chief of this journal.

## Competing interests

The authors declare that they have no competing interests.

## Authors’ contributions

ROR assessed both twins in 2010 and obtained informed consent for publication. EFT assessed both twins in 1988. JR reviewed the 2010 videotapes of the interviews to confirm diagnosis. SS co‒authored the paper. All authors read and approved the final manuscript.
